# The association of RBX1 and BAMBI gene expression with oocyte maturation in PCOS women

**DOI:** 10.1186/s12920-024-01800-2

**Published:** 2024-01-18

**Authors:** Kimia Monshizadeh, Masoud Tajamolian, Fatemeh Anbari, Mohammad Yahya Vahidi Mehrjardi, Seyed Mehdi Kalantar, Mohammadreza Dehghani

**Affiliations:** 1 Department of Medical Genetics, Shahid Sadoughi University of Medical Sciences, Yazd, Iran; 2 Medical Genetics Research Center, Shahid Sadoughi University of Medical Sciences, Yazd, Iran; 3 Research and Clinical Center for Infertility, Yazd Reproductive Sciences Institute, Shahid Sadoughi University of Medical Sciences, Yazd, Iran; 4 Department of Reproductive Biology, Shahid Sadoughi University of Medical Sciences, Yazd, Iran; 5 Research Center for Food Hygiene and Safety, School of Public Health, Shahid Sadoughi University of Medical Sciences, Yazd, Iran

**Keywords:** *BAMBI*, Cumulus cells, Oocyte maturation, Polycystic ovarian syndrome, *RBX1*

## Abstract

**Background:**

Polycystic ovarian syndrome (PCOS) is a common endocrine disorder that affects 6–20% of women of reproductive age. One of the symptoms of PCOS is hyperandrogenism, which can impair follicular development. This disruption can cause issues with the development of oocytes and the growth of embryos. Although the exact cause of PCOS is not yet fully understood, studying the gene expression pattern of cumulus cells, which play a crucial role in the maturation and quality of oocytes, could help identify the genes associated with oocyte maturation in PCOS women. Through indirect activation of APC/Cdc20, *RBX1* enables oocytes to bypass the GV (germinal vesicles) stage and advance to the MII (metaphase II) stage. our other gene is the *BAMBI* gene which stimulates WNT signaling, that is a crucial pathway for healthy ovarian function. This study aims to explore the expression level of the *RBX1* and *BAMBI* genes between GV and MII oocytes of PCOS and non-PCOS groups.

**Methods:**

In this experiment, we gathered the cumulus cells of MII (38 cases and 33 control) and GV (38 cases and 33 control) oocytes from women with/without PCOS. Besides, quantitative RT-PCR was used to assess the semi-quantitative expression of *BAMBI* and *RBX1*.

**Results:**

According to our research, the expression level of *RBX1* and *BAMBI* in MII and GV cumulus cells of PCOS patients was significantly lower than that in non-PCOS ones.

**Conclusion:**

This research raises the possibility of *RBX1* and *BAMBI* involvement in oocyte quality in PCOS women.

## Introduction

Polycystic ovarian syndrome (PCOS) affects six to twenty percent of reproductive-age females. This syndrome, which is identified by the presence of at least two out of the three distinctive features of Rotterdam criteria including hyperandrogenism, oligo-anovulation, and polycystic ovaries [1], is one of the most common endocrine conditions in women. Additionally, there are clear connections between PCOS and other metabolic illnesses, including cancer, hypertension, diabetes mellitus, and cardiovascular diseases [2]. Metabolic dysfunction in PCOS patients causes issues with oocyte maturation and embryonic development, which is caused by the change in the intrafollicular microenvironment during folliculogenesis [3].

The cumulus cells are located close to the oocyte and maintain communication with it by transzonal and gap junctions [4]. In the cumulus-oocyte complex (COC), there is a bidirectional relationship between the oocyte and the cumulus, leading to oocyte maturation as well as cumulus expansion and differentiation. During the maturation of the oocyte, cumulus cells provide a gateway for the transfer of various substances, including nutrients, regulatory molecules, and paracrine factors [5]. Furthermore, oocytes release substances that promote the differentiation and growth of cumulus cells [6]. Evidence suggests that cumulus cells have an important role in oocyte quality, fertilization, as well as early embryonic development, and pregnancy. Therefore, investigating the expression profile of cumulus cells is a non-invasive way to evaluate the quality of oocytes [7, 8]. There have been several attempts to figure out the gene expression profile of cumulus cells by microarray and RNA sequencing.

 The Wnt/β-catenin signaling can influence the activation of the female reproductive system by altering the hormonal activity in ovarian granulosa cells [22]. Among the genes that have the ability to favorably activate this pathway is *BAMBI* [10]. Numerous investigations have demonstrated the critical function the SCF complex plays in oogenesis [9]. The catalytic core of SCF, a multi-subunit proteasome, is formed by *RBX1*, one of its essential constituents [10]. Indirect activation of APC/Cdc20 by *RBX1* can cause an oocyte to bypass the GV stage and advance to the MII stage [11, 12]. This study aimed to determine if these genes are involved in oocyte maturation, taking into account their role in ovulation.

## Methods

### Participants’ characteristics and experimental design

Participants who met the Rotterdam criteria for polycystic ovarian syndrome were the subjects of this investigation. They were infertile and referred to Yazd Reproductive Sciences Institute for treatment. Clinical information was gathered for all the study subjects. The exclusion criteria were androgen-secreting tumors, congenital adrenal hyperplasia, and Cushing’s syndrome. Cumulus cell samples were collected from the PCOS and non-PCOS women. The non-PCOS group included people who did not have PCOS or any other cause of female infertility and underwent ICSI treatment for male infertility. We considered non-PCOS women as a control group. Based on the examination of the oocyte nucleus, the maturity stage was determined. The oocytes with an extruded polar body were considered mature (MII), but those with a fully developed germinal vesicle were considered immature (GV). The cumulus cells (CCs) were isolated from COCs at the GV (GV-CC) and MII (MII-CC) stages. We collected 38 MII-CC and 38 GV-CC for the PCOS group. Also, 33 MII-CC and 33 GV-CC were gathered from non-PCOS women.

### Oocyte retrieval and isolation of CCs

Oocyte retrieval was carried out using ultrasonography 35–36 h following HCG injection, and the COCs used in this investigation were identified with a stereomicroscope. Mechanical and enzyme methods were used to denude the CCs and oocytes from the COCs complex. After the CCs were separated from the COCs, the resulting CCs were washed with PBS. CCs were transferred to − 80 °C in a TRIzol® Reagent (Yekta Tajhiz Azuma, Iran) to preserve RNA until the next step was initiated.

### RNA extraction and cDNA synthesis

TRIzol® Reagent was used to extract the total RNA from the samples. The concentration and purity of RNA were also determined with a NanoDrop spectrophotometer (DeNovix, USA), the results were verified by agarose gel electrophoresis, and then the extracted RNA was immediately used in the next step. Based on the manufacturer’s instructions provided by the Parstous Easy cDNA Synthesis Kit (Parstous, Iran), cDNA was synthesized for both *BAMBI* and *RBX1* genes.

### Quantitative real-time PCR (qPCR)

The confirmation of the selected genes was carried out using quantitative-PCR (q-PCR). The sequences of the primers utilized in this study are presented in Table 1. With the use of the SYBR Green master mix (Yekta Tajhiz Azuma, Iran) and the Rotor Gene-Q device (Qiagen, Germany), the q-PCR reaction was carried out to ascertain the relative expression of the genes. To eliminate manual mistakes, all reactions were done in duplicate. The initial denaturation was conducted for RT-PCR at 95 °C for 5 min followed by 45 cycles of denaturation at 95 °C for 20 s, annealing at 61 °C for 20 s, and extension at 72 °C for 30 s. As an endogenous control of normalization, we used the *ACTB* housekeeping gene. We used *ACTB* gene as the reference because according to Jesús Cadenas and colleagues (2022), *ACTB* is a suitable reference gene for the study of cumulus cells and oocytes [13].

**Table 1:** The list of primers was employed in this research project

Primer	Sequences	Annealing temperature (°C)	Amplicon size (bp)
F-RBX1	AACTGTGCCATCTGCAGGAA	59.89	170
R-RBX1	TCCAATGGACACACCTGTCG	59.97	
F-BAMBI	CGCCACTCCAGCTACATCTT	59.82	104
R-BAMBI	CAGTGGGCAGCATCACAGTA	60.04	
F-ACTB	GGGAAATCGTGCGTGACATT	59.20	183
R-ACTB	GGAAGGAAGGCTGGAAGAGT	59.01	

qPCR: Quantitative polymerase chain reaction, *RBX1*: Ring box protein 1, *BAMBI*: Bone morphogenetic protein and activin membrane-bound inhibitor, ACTB: β-Actin, bp: Base pairs, F: Forward, R: Reverse

### Statistical analysis

GraphPad Prism, version 8.4.3, and IBM SPSS Statistics, version 26 (SPSS Inc., Chicago, USA), were used to analyze the data. The analysis was done through one-way ANOVA and multiple t-tests to check whether there was a significant difference between the groups. The statistical significance was determined by P-values less than 0.05.

## Results

Table 2 reports the clinical data of the women with PCOS and those without PCOS who took part in this study. The result, as shown in Table 2, indicates that no significant difference between age, BMI, and FSH ratio in the two groups. The women with PCOS displayed a greater ratio of basic LH (9.1 ± 1.6) and LH/FSH (1.38 ± 0.6) than the non-PCOS women with LH (4.1 ± 1.5) and FSH (0.6 ± 0.4). They achieved these values at *P* < 0.05 and *P* < 0.001, respectively.

**Fig. 1:**
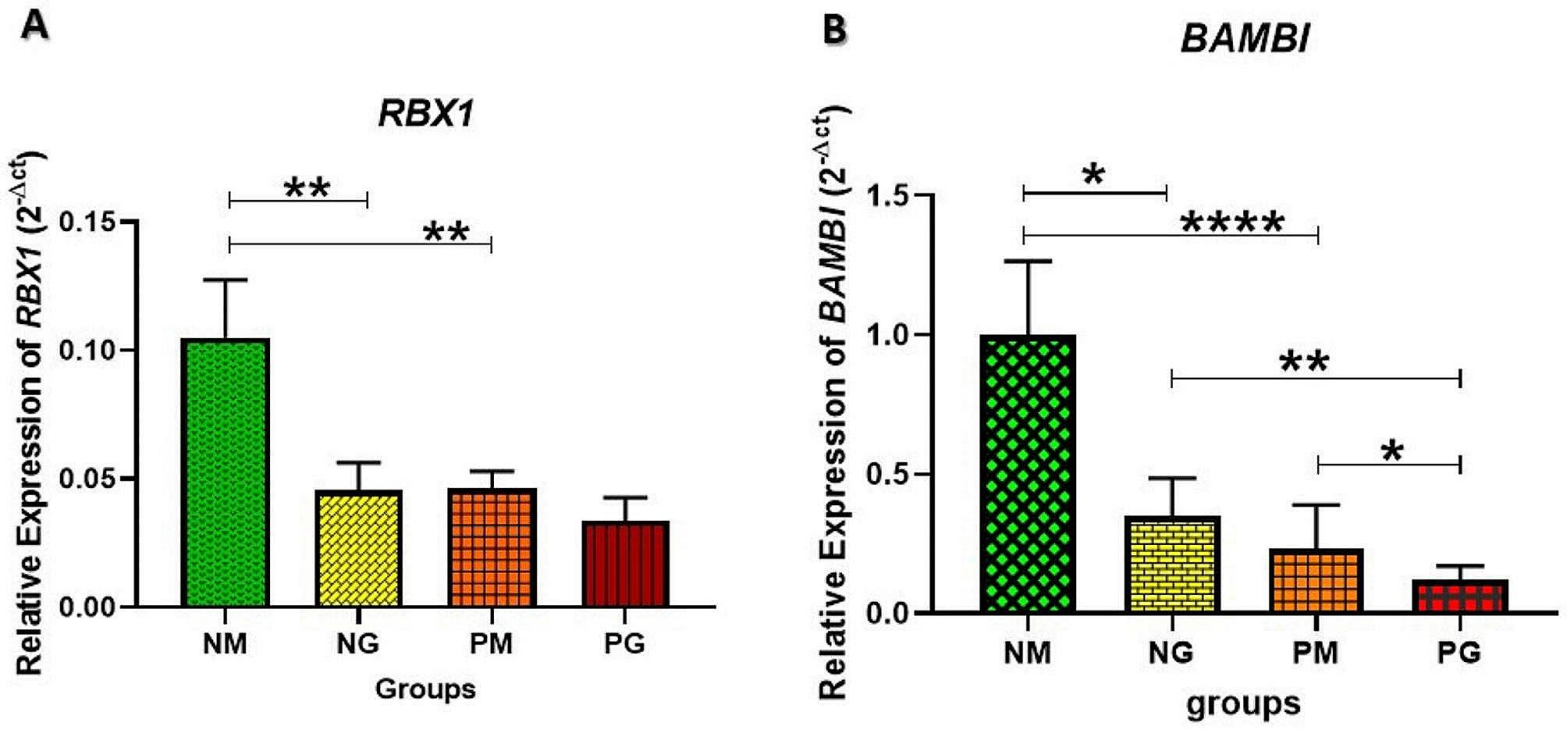
The gene expressions of *RBX1* and *BAMBI* in CCs with and without PCOS at GV and MII stages. *Indicates a significant difference in gene expression. *PM*: PCOS at MII stage, *PG*: PCOS at GV stage, *NM*: non-PCOS at MII stage, *NG*: non-PCOS at GV stage. (**a**) The relative expression of the *RBX1* gene in PCOS and non-PCOS groups based on the developmental stages of the oocyte. (**b**) The expression of the *BAMBI* gene between groups with different oocyte maturation stages

When compared to the non-PCOS patients, the PCOS women emerged to have a significantly lower expression of *RBX1* gene in their CCs at the MII stage (*P* = 0.0019). Furthermore, the expression of *RBX1* gene in the CCs of the non-PCOS women at the GV stage was significantly lower than that of the non-PCOS women at the MII stage (*P* = 0.0024) (Fig. 1a). The expression of *BAMBI* gene significantly was lower in the CCs of the PCOS patients at both the GV (*P* = 0.02) and the MII (*P* < 0.0001) stages, compared to that of the non-PCOS women. Additionally, the expression of *BAMBI* gene in the CCs of the PCOS women at the GV stage had significantly decreased compared to that in the CCs of the PCOS patients at the MII stage (*P* = 0.023). The expression of this gene was significantly lower in the CCs of the non-PCOS women at both stages (*P* = 0.011) (Fig. 1b).

**Table 2:** Clinical characteristics of women with and without PCOS

Parameters	Non-PCOS	PCOS	P-value
Age (year)	34.50 ± 4.00	32.58 ± 5.48	0.46
BMI (kg/m2)	26.55 ± 3.94	28.16 ± 5.53	0.38
FSH (IU/I)	6.8 ± 1.2	6.6 ± 1.8	0.25
LH (IU/I)	4.1 ± 1.5	9.1 ± 1.6	< 0.05
LH/FSH	0.6 ± 0.4	1.38 ± 0.6	< 0.001

Non-PCOS vs PCOS group. BMI: Body Mass Index. FSH: Follicle Stimulating Hormone. LH: Luteinizing Hormone. The values were obtained using a t-test and the results are reported as mean ± SD. *p < 0.05, **p < 0.001

## Discussion

E3 ligases serve to catalyze the transfer of ubiquitin to a particular substrate, which completes the ubiquitination process [14]. One of the common families of E3 enzymes is the Skp1-cullin 1-F-box (SCF) complex, which is made up of three invariant components including Cullin (*Cul*), Ring box protein 1 (*RBX1*), and S-Phase Kinase Associated Protein 1(*Skp1*). The cataleptic core of the SCF complex is made up of *RBX1* and *Cul1* [15].

Yi and colleagues [16] showed that the inhibition of the proteasome complex keeps oocytes at the GV stage. A different morphology was observed by Kinterova and colleagues [12] for cumulus cells from oocytes matured in culture media containing an inhibitor of the SCF complex, which may cause problems during the expansion of cumulus cells. Cumulus expansion is an essential process involved in ovulation, oocyte maturation, and sperm capacitation. When cumulus cells expand, a change occurs in the junctional communication between them and oocytes, thus contributing to oocyte maturation [17]. Studies have shown that the SCF complex is essential for the proper maturation of oocytes, cumulus cell expansion, spermatogenesis, embryogenesis, and fertilization [12].

*RBX1* is an important component of the SCF complex that degrades Early mitotic inhibitor 1 *(EMI)*. EM*I* is an inhibitor of the anaphase-promoting complex/cyclosome (*APC/C*). Inhibition of this complex prevents the degradation of cyclin B and securin, which leads to stopping oocytes from progressing to the MII stage. Destruction of *EMI* by *RBX1* activates APC/cdc20, allowing for chromosome separation and anaphase onset. Dysregulation of *RBX1* can result in the accumulation of *EMI* and consequent inhibition of APC/cdc20, leading to anaphase arrest and keeping the cells in the GV stage [18].

In the present study, through a comparison of the MII-CCs in the PCOS patients were found to have lower gene expression than those of the non-PCOS. The expression of *RBX1* was also discovered to be lower in the CCs of the non-PCOS patients at the GV stage than in the MII stage. However, there was no significant difference in expression between the GV and MII stages in the PCOS group. This finding suggests that *RBX1* may play a role in improving the quality of maturation. As Zhou and colleagues [18] found that *RBX1* has an indispensable role in mouse oocyte meiotic maturation, embryonic development, and cell proliferation. To find further evidence, the outcomes were followed for these groups. In some cases, the mature oocytes taken from the PCOS women who underwent IVF failed to fertilize successfully and resulted in the loss of embryos, as opposed to the mature oocytes taken from non-PCOS women. This evidence, along with the molecular function of *RBX1*, can explain how this gene plays a role in oocyte quality in PCOS.

The WNT signaling pathway plays an imperative role in normal ovarian function, fertility [9, 19], and development of follicles [9, 20], as well as during embryogenesis [21]. Akino and colleagues [22] showed that the dysregulation of this pathway in cumulus cells can result in follicle atresia, a reduced fertilization rate, and abnormal embryogenesis. The Wnt/β-catenin pathway is initiated when a ligand binds to the low-density lipoprotein receptor-related protein 5/6 (LRP5/6) and the Frizzled receptor. Without a suitable ligand, β-catenin is degraded by a destructive complex made up of GSK3B, Adenomatous Polyposis Coli (*APC*), and Axin [20]. However, when a Wnt ligand attaches to its receptor, the destructive complex stops and β-catenin accumulates in the cytoplasm and is transported into the nucleus [23].

Bone morphogenetic protein and activin membrane-bound inhibitor (*BAMBI*) is shown to interact with Frizzled5 and Dishevelled2 receptors and positively activate the Wnt/β-catenin pathway. *BAMBI* knockdown impairs the Wnt signaling pathway, whereas its overexpression enhances cell cycle progression and transition from G1 to S phase by activating beta-catenin nuclear translocation and stimulating cyclin D1 and c-myc synthesis [24, 25].

Using microarray, Assou and colleagues (2006) showed that the *BAMBI* gene is overexpressed in human cumulus cells. They stated that the overexpression of the genes having a role in cell-to-cell communication helps the maturation of the oocyte-cumulus complex [26]. Xinjian Li and colleagues (2020) performed high-throughput sequencing to identify candidate genes for reproductive traits in pigs. Their findings revealed that the *BAMBI* gene is related to reproductive traits [27]. Long Bai and colleagues (2014) examined the role of *BAMBI* in porcine granulosa cells, finding that this gene can regulate the development of ovarian follicles and the maturation of oocytes [28]. Lankford and colleagues [29] found that the relative expression of *BAMBI* gene was increased in the follicles that were competent to respond to the maturation-inducing hormone and also in mature follicles suggesting that *BAMBI* may contribute to the maturation process.

In the present study, it was discovered that the expression of the *BAMBI* gene was down-regulated in the MII-CC of the PCOS compared to MII-CC of the non-PCOS groups, and this decrease in *BAMBI* expression in PCOS women can result in an arrested cell cycle in the primary stage, which may be the cause of lower oocyte quality in those patients. The result of the normal groups at various stages of differentiation revealed that this gene might be involved in the maturation process because its expression was downregulated in the GV-CC group compared to the MII-CC group. Furthermore, a comparison of immature cumulus cells to mature cumulus cells showed a downregulation of expression among PCOS women. It seems that this gene may contribute to oocyte maturation.

## Conclusion

Gene expression analysis of cumulus cells provides a non-invasive method for examining oocyte conditions. According to our research, *RBX1* and *BAMBI* may be involved in oocyte quality in PCOS patients; however, more research is needed to validate this claim.

## Data Availability

The data that support the findings of this study are available from the corresponding author, upon reasonable request.

## Abbreviations

PCOS: polycystic ovarian syndrome
RBX1: Ring box protein 1
APC/Cdc20: anaphase-promoting complex/cyclosome
GV: germinal vesicles
MII: metaphase II
WNT: Wingless/Integrated signaling pathway
BAMBI: Bone morphogenetic protein and activin membrane-bound inhibitor
RT-PCR: Reverse transcription polymerase chain reaction
COC: cumulus-oocyte complex
SCF complex: Skp, Cullin, F-box containing complex
ICSI: Intracytoplasmic sperm injection
CCs: cumulus cells
GV-CC: COCs at the GV
MII-CC: COCs at the MII
HCG: Human chorionic gonadotropin
PBS: Phosphate-buffered saline
ACTB: Actin beta
BMI: Body Mass Index
FSH: Follicle Stimulating Hormone
LH: Luteinizing Hormone
NM: non-PCOS at MII stage
NG: non-PCOS at GV stage
PM: PCOS at MII stage
PG: PCOS at GV stage
IVF: In vitro fertilization

## References

[1] Alvandian F, Hosseini E, Hashemian Z, Khosravifar M, Movaghar B, Shahhosein M (2022). TGFß gene members and their Regulatory factors in Granulosa compared to Cumulus cells in PCOS: a case-control study. Cell J.

[2] Deswal R, Narwal V, Dang A, Pundir CS. The prevalence of polycystic ovary syndrome: a brief systematic review. J Hum Reproductive Sci. 2020. 10.4103/jhrs.JHRS_95_18 PMC7879843 33627974

[3] Dehghan Z, Mohammadi-Yeganeh S, Rezaee D, Salehi M. MicroRNA-21 is involved in oocyte maturation, blastocyst formation, and pre-implantation embryo development. Dev Biol. 2021. 10.1016/j.ydbio.2021.08.008 34411594

[4] Uyar A, Torrealday S, Seli E. Cumulus and granulosa cell markers of oocyte and embryo quality. In: Fertility and Sterility. 2013. 10.1016/j.fertnstert.2013.01.129 PMC3866131 23498999

[5] Shen Q, Chen M, Zhao X, Liu Y, Ren X, Zhang L. Versican expression level in cumulus cells is associated with human oocyte developmental competence. Syst Biol Reprod Med. 2020. 10.1080/19396368.2020.1725685 32138539

[6] Marchais M, Gilbert I, Bastien A, Macaulay A, Robert C. Mammalian cumulus-oocyte complex communication: a dialog through long and short distance messaging. J Assist Reprod Genet. 2022. 10.1007/s10815-022-02438-8 PMC9107539 35499777

[7] Turathum B, Gao EM, Chian RC. The function of cumulus cells in oocyte growth and maturation and in subsequent ovulation and fertilization. Cells. 2021. 10.3390/cells10092292 PMC8470117 34571941

[8] Montazeri F, Kalantar SM, Fesahat F, Sheikhha MH, Omidi M, Shafienia H et al. Association between cumulus cells—mRNA levels of AMHR2 and FSHR with oocyte maturity. Middle East Fertil Soc J [Internet]. 2022;27(1):26. 10.1186/s43043-022-00116-4.

[9] Hernandez Gifford JA. The role of WNT signaling in adult ovarian folliculogenesis. Reproduction. 2015. 10.1530/REP-14-0685 PMC4560668 26130815

[10] Kim SH, Kim H. Inhibitory effect of astaxanthin on gene expression changes in Helicobacter pylori-infected human gastric epithelial cells. Nutrients. 2021. 10.3390/nu13124281 PMC8708722 34959833

[11] Kinterová V, Kaňka J, Bartková A, Toralová T. SCF Ligases and their functions in Oogenesis and embryogenesis—summary of the most important findings throughout the animal Kingdom. Cells. 2022;11(2). 10.3390/cells11020234 PMC8774150 35053348

[12] Kinterova V, Kanka J, Petruskova V, Toralova T. Inhibition of Skp1-Cullin-F-box complexes during bovine oocyte maturation and preimplantation development leads to delayed development of embryos. Biol Reprod. 2019. 10.1093/biolre/ioy254 30535233

[13] Cadenas J, Pors SE, Nikiforov D, Zheng M, Subiran C, Bøtkjær JA et al. Validating reference gene expression Stability in Human ovarian follicles, oocytes, Cumulus Cells, ovarian Medulla, and ovarian cortex tissue. Int J Mol Sci. 2022. 10.3390/ijms23020886 PMC8778884 35055072

[14] Berndsen CE, Wolberger C. New insights into ubiquitin E3 ligase mechanism. Nat Struct Mol Biology. 2014. 10.1038/nsmb.2780 24699078

[15] Rashpa R, Klages N, Schvartz D, Pasquarello C, Brochet M. The Skp1-Cullin1-FBXO1 complex is a pleiotropic regulator required for the formation of gametes and motile forms in Plasmodium berghei. Nat Commun. 2023. 10.1038/s41467-023-36999-8 PMC10006092 36898988

[16] Yi YJ, Nagyova E, Manandhar G, Procházka R, Sutovsky M, Park CS et al. Proteolytic activity of the 26S proteasome is required for the meiotic resumption, germinal vesicle breakdown, and cumulus expansion of porcine cumulus-oocyte complexes matured in vitro. Biol Reprod. 2008. 10.1095/biolreprod.107.061366 17942798

[17] Robker RL, Hennebold JD, Russell DL. Coordination of ovulation and oocyte maturation: a good egg at the right time. Endocrinology. 2018. 10.1210/en.2018-00485 PMC6456964 30010832

[18] Zhou L, Yang Y, Zhang J, Guo X, Bi Y, Li X et al. The role of RING box protein 1 in mouse oocyte meiotic maturation. PLoS ONE. 2013. 10.1371/journal.pone.0068964 PMC3708900 23874827

[19] Abedini A, Zamberlam G, Lapointe E, Tourigny C, Boyer A, Paquet M et al. WNT5a is required for normal ovarian follicle development and antagonizes gonadotropin responsiveness in granulosa cells by suppressing canonical WNT signaling. FASEB J. 2016. 10.1096/fj.15-280313 PMC4799500 26667040

[20] Mehdinejadiani S, Amidi F, Mehdizadeh M, Barati M, Pazhohan A, Alyasin A et al. Effects of letrozole and clomiphene citrate on wnt signaling pathway in endometrium of polycystic ovarian syndrome and healthy women. Biol Reprod. 2019. 10.1093/biolre/ioy187 30184105

[21] Gonzalez-Ramiro H, Parrilla I, Miquel Cambra J, Gonzalez-Plaza A, Antonia Gil M, Cuello C (2022). Combined synchronization and superovulation treatments negatively impact embryo viability possibly by the downregulation of WNT/β-catenin and notch signaling genes in the porcine endometrium. J Anim Sci.

[22] Akino R, Matsui D, Kawahara-Miki R, Amita M, Tatsumi K, Ishida E et al. Next-generation sequencing reveals downregulation of the wnt signaling pathway in human dysmature Cumulus cells as a Hallmark for evaluating oocyte quality. Reprod Med. 2020.

[23] Liu J, Xiao Q, Xiao J, Niu C, Li Y, Zhang X, et al. Wnt/β-catenin signalling: function, biological mechanisms, and therapeutic opportunities. Signal Transduction and Targeted Therapy; 2022. 10.1038/s41392-021-00762-6 PMC8724284 34980884

[24] Zhao HJ, Chang HM, Klausen C, Zhu H, Li Y, Leung PCK. Bone morphogenetic protein 2 induces the activation of WNT/β-catenin signaling and human trophoblast invasion through up-regulating BAMBI. Cell Signal. 2020. 10.1016/j.cellsig.2019.109489 31786181

[25] Liu K, Song X, Ma H, Liu L, Wen X, Yu J et al. Knockdown of BAMBI inhibits β-catenin and transforming growth factor β to suppress metastasis of gastric cancer cells. Mol Med Rep. 2014. 10.3892/mmr.2014.2305 24912656

[26] Assou S, Anahory T,…VP-H, undefined. The human cumulus–oocyte complex gene-expression profile. 2006. academic.oup.com. 10.1093/humrep/del065 PMC2377388 16571642

[27] Li X, Ye J, Han X, Qiao R, Li X, Lv G et al. Whole-genome sequencing identifies potential candidate genes for reproductive traits in pigs. Genomics. 2020. 10.1016/j.ygeno.2019.01.014 30707936

[28] Bai L, Chu G, Mai Y, Zheng J, Wang W, Zhang Q et al. Identification and expression analyses of BAMBI mediated by FSH in swine luteinizing granulosa cells. Theriogenology. 2014. 10.1016/j.theriogenology.2014.07.022 25168722

[29] Lankford SE, Weber GM. Temporal mRNA expression of transforming growth factor-beta superfamily members and inhibitors in the developing rainbow trout ovary. Gen Comp Endocrinol. 2010. 10.1016/j.ygcen.2009.09.007 19781545

